# Prevalence and risk factors for neonatal sepsis among very preterm infants in China: a systematic review and meta-analysis

**DOI:** 10.3389/fped.2026.1815128

**Published:** 2026-04-01

**Authors:** Yuanyuan Li, Xin Guo, Kanghua Zhou, Jing Feng, Dandan Rao, Guilian Du, Zhangbin Yu, Huaiwu Zheng

**Affiliations:** 1 Neonatology, The People’s Hospital of Baoan Shenzhen, Shenzhen, Guangdong, China; 2 Neonatology, Longgang District Maternity & Child Healthcare Hospital of Shenzhen City (Longgang Maternity and Child Institute of Shantou University Medical College), Shenzhen, Guangdong, China; 3 Neonatology, Shenzhen Baoan Women’s and Children’s Hospital, Shenzhen, Guangdong, China; 4 Neonatology, Shenzhen Luohu People’s Hospital, Shenzhen, Guangdong, China; 5 Neonatology, Shenzhen Bao'an District Songgang People’s Hospital, Shenzhen, Guangdong, China; 6 Department of Neonatology, Shenzhen People’s Hospital, The Second Clinical Medical College, Jinan University; The First Affiliated Hospital, Southern University of Science and Technology, Shenzhen, Guangdong, China

**Keywords:** China, prevalence, risk factors, sepsis, very preterm infants

## Abstract

**Background:**

Neonatal sepsis poses a significant risk to very preterm infants (VPIs, gestational age < 32 weeks) in China, with limited nationwide data on its incidence and risk factors. This study aimed to address this gap through a systematic review and meta-analysis to inform prevention strategies.

**Methods:**

We searched PubMed, Embase, Web of Science, Scopus, CNKI, CBM, CSTJ, and WanFang up to March 31, 2025. Study quality was evaluated using the Newcastle-Ottawa Scale (NOS). Data extraction was performed with Microsoft Excel, and Stata 18.0 was used for meta-analysis. Heterogeneity was assessed using Cochran's *Q* and *I*^2^ statistics, while publication bias was evaluated with funnel plots and Egger's test. Subgroup analyses were conducted to identify sources of heterogeneity.

**Results:**

This systematic review encompassed 43 studies involving a total of 138,613 VPIs. The incidence of unclassified sepsis was determined to be 16.41% (95% CI: 11.80%–21.62%), while the incidence rates for early-onset sepsis (EOS) and late-onset sepsis (LOS) were 16.11% (95% CI: 9.52%–24.01%) and 15.10% (95% CI: 12.07%–18.40%), respectively. The study identified six significant risk factors for EOS among VPIs: lower gestational age [Hedges’ *g* = −0.82, 95% CI: (−1.12, −0.52)], lower birth weight [Hedges’ *g* = −0.46, 95% CI: (−0.64, −0.27)], chorioamnionitis (OR 3.10, 95% CI: 2.72–3.54), premature rupture of membranes (PROM) (OR 1.51, 95% CI: 1.12–2.04), administration of antenatal antibiotics (OR 1.38, 95% CI: 1.22–1.56), and the requirement for endotracheal intubation (OR 5.87, 95% CI: 3.84–8.97).

**Conclusions:**

Sepsis imposes a substantial burden on Chinese VPIs, characterized by a highly heterogeneous incidence. The principal risk factors for EOS include lower gestational age, lower birth weight, chorioamnionitis, PROM, antenatal antibiotic administration, and endotracheal intubation. These findings furnish essential evidence for the development of risk prediction models and stratified management strategies for EOS in VPIs.

**Systematic trial registration:**

The systematic review and meta-analysis were registered with PROSPERO (ID: CRD420251018993).

## 1 Introduction

Neonatal sepsis (NS) is among the most prevalent and severe infectious diseases affecting newborns, significantly contributing to neonatal morbidity and mortality (1–3). A comprehensive systematic review of multiple observational epidemiological studies has determined that the global incidence rate of neonatal sepsis is 2,202 cases per 100,000 live births, with a case fatality rate between 11% and 19% (4).

China has achieved substantial advancements in the care of very preterm infants (VPIs) (gestational age < 32 weeks), with survival rates improving markedly from 69.2% in 2010 (5) to 87.6% in 2019 (6). Despite these advancements, sepsis continues to pose a significant clinical challenge for these high-risk neonates, as it is associated with increased morbidity and mortality, as well as poorer outcomes among survivors (7). Notably, the reported incidence of neonatal sepsis varies considerably across different regions and healthcare facilities in China, with figures ranging from 1% (8) to 45% (9). At present, there is a significant deficiency in comprehensive nationwide epidemiological data and systematic analyses of risk factors pertaining to VPIs in China, underscoring an urgent need for advancements in this domain. Early identification of these risk factors has the potential to reduce healthcare expenditures by diminishing the necessity for costly treatments and extended hospital stays.

In light of these identified gaps, this study seeks to perform a systematic review and meta-analysis to estimate the aggregated incidence of sepsis and determine the associated risk factors among VPIs in China. This research aims to furnish evidence that will inform the development of targeted prevention and treatment strategies.

## 2 Methods

The systematic review and meta-analysis followed the PRISMA reporting guideline (10) and were registered with PROSPERO (registration number: CRD420251018993).

### 2.1 Search strategy

We conducted a comprehensive search of the PubMed, Embase, Web of Science, Scopus, China National Knowledge Infrastructure (CNKI), Chinese Biomedical Literature Database (CBM), China Science and Technology Journal Database (CSTJ), and WanFang databases from their inception until March 31, 2025. Our search strategy employed a combination of keywords, Medical Subject Headings (MeSH) terms, and their synonyms pertinent to “preterm infant,” “sepsis,” and “China.” The detailed search methodology is provided in Supplementary Material. All identified studies published in English or Chinese were imported into EndNote reference management software for subsequent screening and evaluation.

### 2.2 Definition

Definitions of sepsis vary across studies and encompass criteria such as the Neonatal Sepsis Diagnostic Criteria (2003 Edition) (11), the Expert Consensus on Diagnosis and Treatment of Neonatal Sepsis (2019 Edition) (12) or the 4th/5th editions of Practical Neonatology (13). Additionally, studies utilizing sepsis-relevant ICD codes were included. According to the Expert Consensus on Diagnosis and Treatment of Neonatal Sepsis (2019 Edition) (12), clinical sepsis is characterized by the presence of clinical abnormalities in conjunction with at least one of the following criteria: (a) two or more positive nonspecific blood tests, include white blood cell (WBC), immature to total neutrophil ratio (I/T), platelet count (PLT), C-reactive protein (CRP), and procalcitonin (PCT); (b) cerebrospinal fluid findings indicative of purulent meningitis; or (c) identification of pathogenic bacterial DNA in blood or cerebrospinal fluid. Culture-proven sepsis is conclusively diagnosed when clinical manifestations are accompanied by a positive culture from blood or cerebrospinal fluid. Some studies did not differentiate between culture-proven and clinically diagnosed cases, instead presenting aggregated data. Unless specified otherwise, the incidence of sepsis reported in this study pertains to the combined data of these two categories. For clinical classification, cases are designated as early-onset sepsis (EOS) if they occur within 72 h post-birth, and as late-onset sepsis (LOS) if they occur after 72 h (12). Studies without onset data were categorized as unclassified sepsis.

### 2.3 Inclusion and exclusion criteria

Studies were included in the meta-analysis if they met the following criteria: (a) Geographic scope: Studies conducted in China. (b) Population: Very preterm infants (gestational age < 32 weeks). (c) Study design: Observational studies (including retrospective and prospective cohort studies, cross-sectional, and case‒control designs). (d) All eligible studies reporting either the incidence of neonatal sepsis or risk factors associated with neonatal sepsis data were included. (e) Language: Peer-reviewed publications in English or Chinese. Studies were excluded if they met any of the following criteria: (a) Repeatedly published studies or studies potentially utilizing duplicate data. (b) Abstracts, clinical trial registries, and medical records. (c) Conference proceedings, review articles, letters, and editorials. (d) Incomplete data or inability to extract relevant data.

### 2.4 Study selection

The databases were screened by two independent researchers. Articles were selected based on their titles and abstracts. All duplicate studies were removed. For those that were potentially eligible, the full texts were read. Studies that did not meet the necessary criteria for inclusion at this stage were also excluded from the research. Finally, the information obtained from the remaining studies was measured and extracted. To minimize errors and bias, all the above steps and the evaluation of the methodological quality of each paper were carried out separately by the two independent researchers. If the two researchers disagreed, a third researcher conducted the related evaluations.

### 2.5 Data extraction

Data from the selected studies were separately extracted via a predesigned Microsoft Excel 2021 spreadsheet. The spreadsheet included the first author's name, year of publication, date of investigation, study area, study design, sample size, sample sources, number and rate of sepsis, sepsis type, definition, and potential risk factors for sepsis. For each risk factor, adjusted or unadjusted odds ratios (ORs) were recorded when available. ORs and 95% confidence intervals (CIs) were calculated with a 2 × 2 table using the number of patients with and without a given risk factor who developed neonatal sepsis. For continuous variables, the mean ± standard deviation was calculated when available. If not available, the sample mean and standard deviation were estimated using the validated method by Wan et al. (14) based on median, interquartile range and sample size. Any discrepancies between the data extractors were resolved through discussion and re-evaluation of the studies.

### 2.6 Quality assessment

Two independent reviewers performed the quality assessment. The Newcastle-Ottawa Scale (NOS) was used to assess the quality of the included cohort and case-control studies (15). The quality of the cross-sectional studies was assessed via the standardized Joanna Briggs Institute (JBI) critical appraisal tool (16). Any disagreements between the two quality reviewers were handled by repeating the procedures and involving a third reviewer before the final appraisal results were computed.

### 2.7 Statistical methods and analysis

The extracted data were analyzed using Stata 18.0. Publication bias was assessed through funnel plot visualization and Egger's regression asymmetry test, while heterogeneity was evaluated using Higgins’ *I*^2^ statistics (with *I*^2^ > 50% indicating substantial heterogeneity) and Cochran's *Q* test (with *P* < 0.10 considered statistically significant). The studies were stratified by sepsis type (EOS, LOS, or unclassified sepsis). Given the anticipated clinical and methodological heterogeneity, pooled prevalence estimates were calculated using a DerSimonian-Laird random effects model. Sensitivity analysis was conducted using the leave-one-out method. To explore sources of heterogeneity, subgroup analyses were performed based on geographical setting, sample size, sample sources, and method of diagnosis. Effect sizes are reported as odds ratios (ORs) with 95% confidence intervals (CIs) for dichotomous outcomes and Hedges’ *g* (bias-corrected standardized mean difference) with 95% CIs for continuous outcomes. As this systematic review focused on descriptive synthesis, a formal assessment of the certainty of evidence was not conducted.

## 3 Results

### 3.1 Study selection

**Figure 1:**
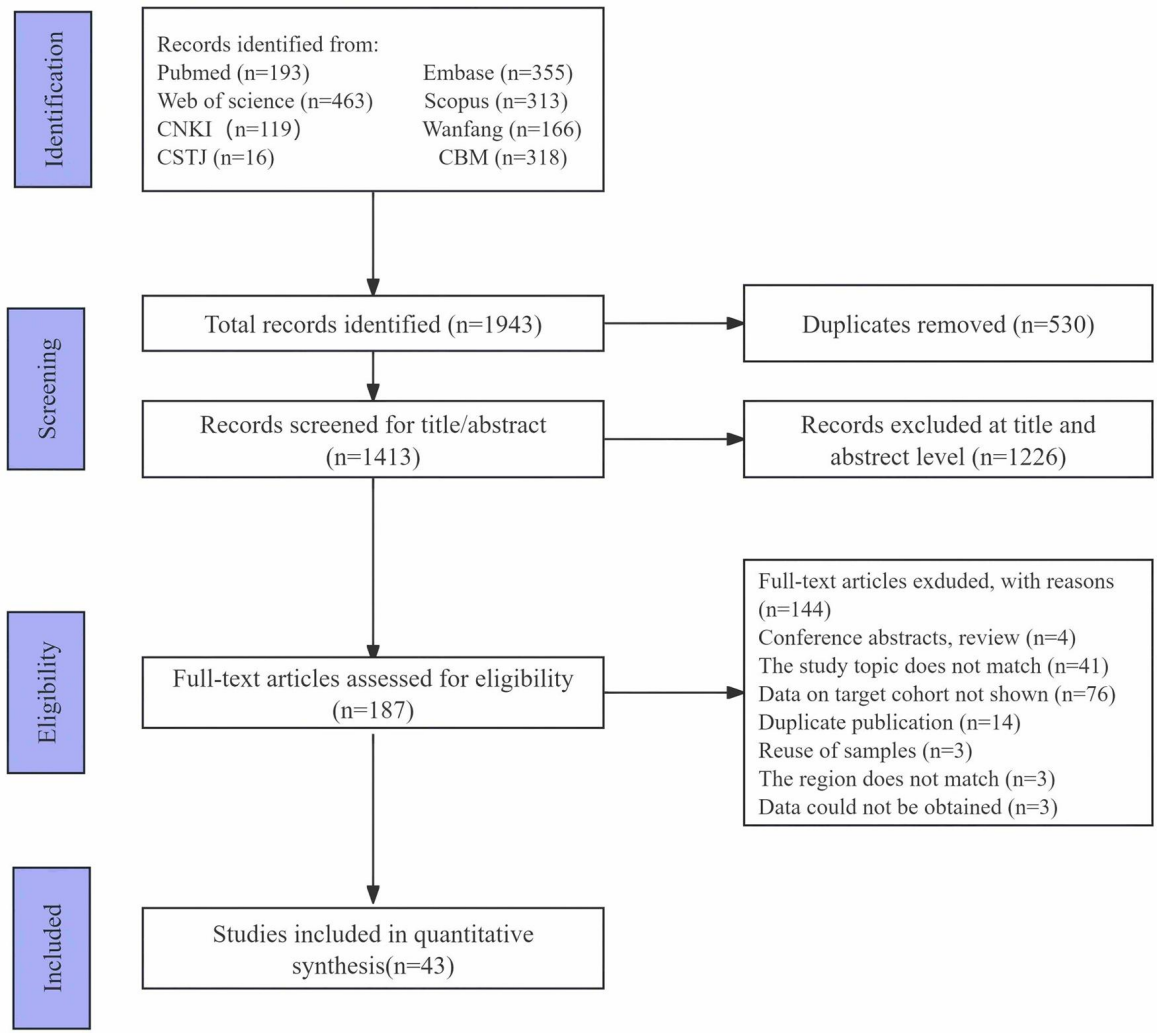
PRISMA flow diagram of the literature search.

A total of 1,943 articles were retrieved from eight databases. Of these, 530 duplicate studies were removed. Following a review of the titles and abstracts, 1,226 articles were excluded. Subsequently, the full texts of potentially relevant articles were assessed for eligibility based on predefined criteria, resulting in the exclusion of 144 articles for various reasons. Ultimately, 43 articles (6–9, 17–55) met the inclusion criteria (Figure 1).

### 3.2 Study characteristics

Table 1 presents the fundamental characteristics of the studies included in this analysis. The articles were published between 2014 and 2025. Among these, 36 were retrospective cohort studies (6–9, 17–19, 21–24, 26, 29–35, 37, 39–43, 45–55), 4 were prospective cohort studies (27, 28, 36, 38), 2 were cross-sectional studies (20, 44), and 1 was a case-control study (25). Additionally, 22 studies were conducted across multiple centers (6–8, 17–20, 22–24, 28, 32–34, 36, 41–44, 46, 49, 52), while 21 were single-center studies (9, 21, 25–27, 29–31, 35, 37–40, 45, 47, 48, 50, 51, 53–55). The sample sizes varied from 21 (37) to 17,874 (23), with a cumulative total of 138,613 live births. Eighteen studies reported on unclassified sepsis incidence (6, 7, 18, 19, 22, 26, 34, 40, 41, 43, 44, 48–50, 52–55), 14 studies provided data on both early-onset sepsis (EOS) and late-onset sepsis (LOS) incidence (8, 9, 20, 21, 28, 29, 32, 33, 37, 38, 42, 45–47), 5 studies exclusively reported EOS incidence (23, 24, 30, 36, 39), and 3 studies focused solely on LOS incidence (17, 27, 35). Three studies did not include incidence data and were used exclusively for analyzing risk factors (25, 31, 51). Twenty-four studies reported sepsis-related risk factors, including 15 studies on EOS (8, 20, 21, 24, 25, 28, 30, 32, 35–39, 41, 50), 9 on LOS (8, 21, 27, 28, 32, 37, 38, 41, 51), and 8 on unclassified sepsis risk factors (7, 18, 19, 22, 26, 31, 34, 48).

**Table 1:** Summary characteristics of 43 studies.

Author	Publication years	Province, area	Study design	Sample sources	Sample size	Sepsis rate (%)	Sepsis type	Definition of sepsis	Risk of bias
Li et al. (54)	2014	Beijing (N)	Retrospective cohort study	Single-center	74	32	Unclassified	Undefined	Moderate
Na et al. (55)	2014	Zhejiang (S)	Retrospective Case Series	Single-center	135	35.6	Unclassified	Combined sepsis^a^	Moderate
Huang et al. (53)	2016	Beijing (N)	Retrospective cohort study	Single-center	62	16.12	Unclassified	Undefined	High risk
Kong et al. (52)	2016	Beijing (N)	Retrospective cohort study	Multicenter	1,760	9.7	Unclassified	Combined sepsis^a^	Low risk
Zhang et al. (51)	2017	Sichuan (S)	Retrospective cohort study	Single-center	213	NR	EOS	Undefined	Moderate
Zhuang et al. (50)	2017	Hunan (S)	Retrospective cohort study	Single-center	179	29.6	Unclassified	Combined sepsis^a^	Moderate
Wu et al. (49)	2019	Guandong (S)	Retrospective cohort study	Multicenter	1,588	15.7	Unclassified	Culture-proven sepsis	Moderate
Fan et al. (9)	2020	Hubei (S)	Retrospective cohort study	Single-center	389	52.18 (EOS) 11.83 (LOS)	EOS & LOS	Combined sepsis^a^	Low risk
Li et al. (48)	2020	Shandong (N)	Retrospective cohort study	Single-center	150	19.3	Unclassified	Combined sepsis^a^	Moderate
Yan et al. (47)	2020	Jiangxi (S)	Retrospective cohort study	Single-center	55	43.6	Unclassified	Undefined	Moderate
Yu et al. (46)	2020	Shandong (N)	Retrospective cohort study	Multicenter	371	42.59 (EOS) 31.27 (LOS)	EOS & LOS	Combined sepsis^a^	Low risk
Cao et al. (6)	2021	25 provinces (M)	Retrospective cohort study	Multicenter	9,552	9.1	Unclassified	Culture-proven sepsis	Low risk
Duan et al. (44)	2021	Henan (N)	Retrospective cross-sectional study	Multicenter	1,613	15.9	Unclassified	Combined sepsis^a^	Moderate
Pan et al. (43)	2021	18 centers (M)	Retrospective cohort study	Multicenter	12,014	7.4	Unclassified	Combined sepsis^a^	Low risk
SNN (42)	2021	Shandong (N)	Retrospective cohort study	Multicenter	3,659	28.3 (EOS) 13.4 (LOS)	EOS & LOS	Combined sepsis^a^	Low risk
Zhu et al. (41)	2021	31 provinces (M)	Retrospective cohort study	Multicenter	8,259	36.3	Unclassified	Combined sepsis^a^	Low risk
Zhang et al. (45)	2021	Guandong (S)	Retrospective cohort study	Single-center	179	31.8	Unclassified	Combined sepsis^a^	Low risk
Chang et al. (38)	2022	Taiwan (S)	Prospective Cohort Study	Single-center	120	8.33 (EOS) 56.67 (LOS)	EOS & LOS	Undefined	Low risk
Chen et al. (37)	2022	Guandong (S)	Retrospective cohort study	Single-center	21	42.9 (EOS) 15.4 (LOS)	EOS & LOS	Culture-proven sepsis (LOS)	Moderate
Ji et al. (36)	2022	Shandong (N)	Prospective Cohort Study	Multicenter	5,856	1.84	EOS	Culture-proven sepsis	Low risk
Lin et al. (35)	2022	Taiwan (S)	Retrospective cohort study	Single-center	625	12.6	LOS	Undefined	Low risk
Lyu et al. (34)	2022	25 provinces (M)	Retrospective cohort study	Multicenter	6,085	8.6	Unclassified	Culture-proven sepsis	Low risk
Peng et al. (33)	2022	two provinces (M)	Retrospective cohort study	Multicenter	807	1.11 (EOS) 2.43 (LOS)	EOS &LOS	Culture-proven sepsis	Low risk
Shen et al. (32)	2022	Shanghai (N)	Retrospective cohort study	Multicenter	2,514	14.68 (EOS) 13.01 (LOS)	EOS &LOS	Combined sepsis^a^	Low risk
Jue et al. (40)	2022	Henan (N)	Retrospective cohort study	Single-center	1,714	23.28	Unclassified	Combined sepsis^a^	Low risk
Yan et al. (39)	2022	Jiangsu (S)	Retrospective cohort study	Single-center	347	6.34	EOS	Culture-proven sepsis	Low risk
Guo et al. (31)	2023	Anhui (N)	Retrospective cohort study	Single-center	87	NR	Unclassified	Combined sepsis^a^	Moderate
Wei et al. (30)	2023	Henan (N)	Retrospective cohort study	Single-center	344	28.8	EOS	Combined sepsis^a^	Low risk
Zhang et al. (29)	2023	Shanxi (N)	Retrospective cohort study	Single-center	115	44.34 (EOS) 41.74 (LOS)	EOS & LOS	Combined sepsis^a^	Low risk
SNN (28)	2023	6 provinces (N)	Prospective Cohort Study	Multicenter	5,351	35.3 (EOS) 13.9 (LOS)	EOS & LOS	Combined sepsis^a^	Low risk
Chen et al. (23)	2024	CHNN (M)	Retrospective cohort study	Multicenter	17,874	1.46	EOS	Combined sepsis^a^	Moderate
Hong et al. (22)	2024	CHNN (M)	Retrospective cohort study	Multicenter	5,913	9.3	Unclassified	Culture-proven sepsis	Low risk
Lei et al. (21)	2024	Henan (N)	Retrospective cohort study	Single-center	467	15.84 (EOS) 21.22 (LOS)	EOS & LOS	Combined sepsis^a^	Low risk
Li et al. (7)	2024	CHNN (M)	Retrospective cohort study	Multicenter	7,989	9.2	Unclassified	Combined sepsis^a^	Low risk
Lin et al. (20)	2024	SNDN (S)	Retrospective cross-sectional study	Multicenter	683	11.4 (EOS) 4.69 (LOS)	EOS & LOS	Combined sepsis^a^	Moderate
Yuan et al. (8)	2024	CHNN (M)	Retrospective cohort study	Multicenter	9,244	1.4 (EOS) 8.32 (LOS)	EOS & LOS	Culture-proven sepsis	Low risk
Zheng et al. (19)	2024	CHNN (M)	Retrospective cohort study	Multicenter	13,447	7.96	Unclassified	Culture-proven sepsis	Low risk
Fang et al. (27)	2024	Jiangsu (S)	Prospective Cohort Study	Single-center	1,119	8.4	LOS	Culture-proven sepsis	Low risk
Lou et al. (26)	2024	Shandong (N)	Retrospective cohort study	Single-center	225	4.89	Unclassified	Combined sepsis^a^	Moderate
Pang et al. (25)	2024	Jiangsu (S)	Case‒control study	Single-center	170	NR	EOS	Combined sepsis^a^	Moderate
SNN (24)	2024	Shandong (N)	Retrospective cohort study	Multicenter	7,154	14.09	EOS	Combined sepsis^a^	Low risk
Kuo et al. (18)	2025	Taiwan (S)	Retrospective cohort study	Multicenter	8,015	30.36	Unclassified	ICD newborn sepsis	Moderate
Wang et al. (17)	2025	Jiangsu (S)	Retrospective cohort study	Multicenter	2,075	13.01	LOS	Culture-proven sepsis	Low risk

CHNN, Chinese Neonatal Network; EOS, early-onset sepsis; LOS, late-onset sepsis; M, mixed region of China; N, north of China; NR, not reported; S, south of China; SNDN, Shenzhen Neonatal Data Network; SNN, Sino-Northern Neonatal Network.

^a^ The combined data of culture-proven sepsis and clinical sepsis.

### 3.3 Risk of bias in studies

The studies were categorized according to risk-of-bias thresholds: scores of 7 or higher denoted a low risk of bias, scores ranging from 4 to 6 indicated a moderate risk, and scores of 3 or lower signified a high risk. The evaluation demonstrated that, out of the 43 studies analyzed, 27 exhibited a low risk of bias, 15 exhibited a moderate risk, and 1 exhibited a high risk (Table 1).

### 3.4 Meta-analysis

**Figure 2:**
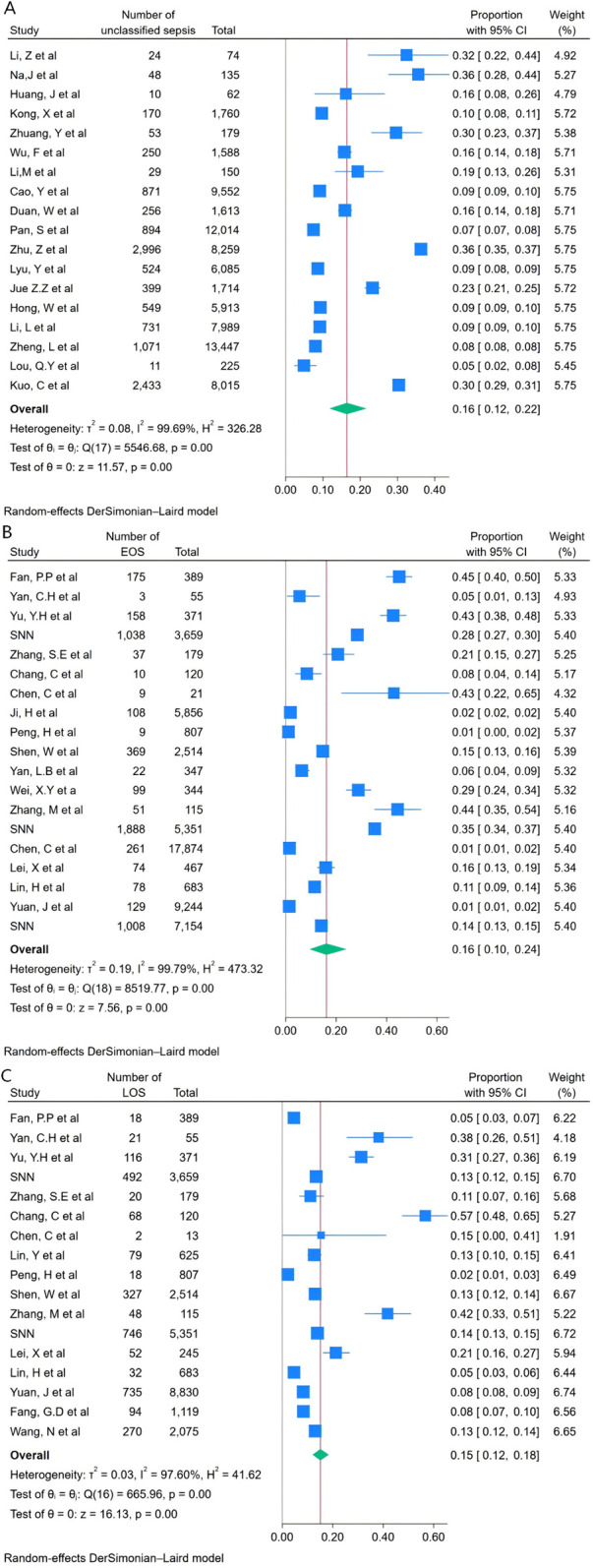
Forest plot of the incidence of unclassified sepsis **(A)**, EOS **(B)**, LOS **(C)**.

**Figure 3:**
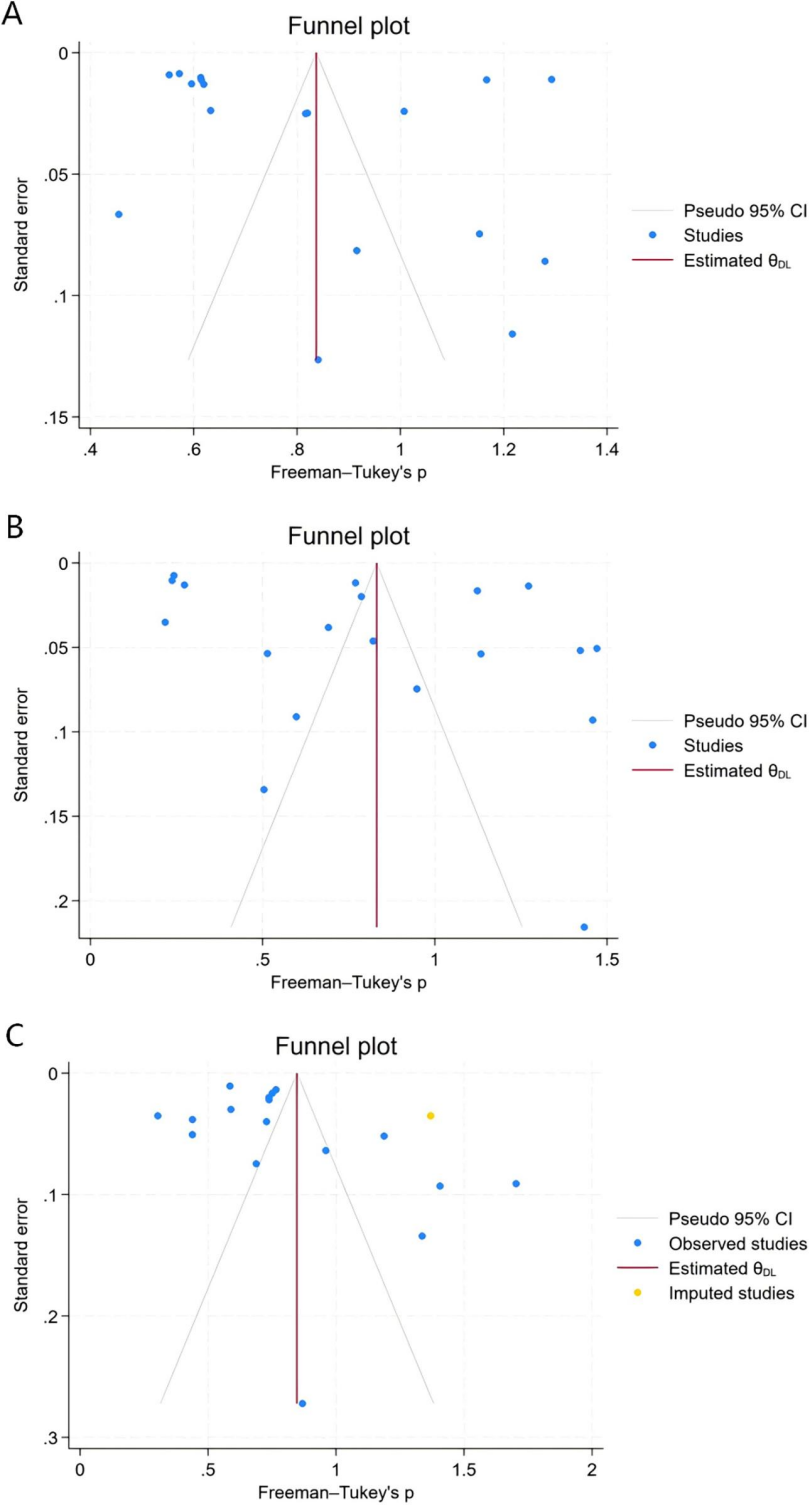
Funnel plot of incidence of unclassified sepsis **(A)**, EOS **(B)** and LOS **(C)**.

The meta-analysis showed that the combined incidence of unclassified sepsis from 18 studies was 16.41% (95% CI: 11.80%–21.62%, *I*^2^ = 99.69 *P* < 0.001) (Figure 2A), while the incidence of EOS from 19 studies was 16.11% (95% CI 9.52%–24.01%, *I*^2^ = 99.79 *P* < 0.001) (Figure 2B), and the LOS from 17 studies was 15.10% (95% CI 12.07%–18.40%, *I*^2^ = 97.60 *P* < 0.001) (Figure 2C). Due to the high heterogeneity, a random effects meta-analysis model was applied to merge the magnitude estimates, as shown in the forest plot (Figure 2). The funnel plot indicated asymmetry in the incidence of unclassified sepsis (Figure 3A) and EOS (Figure 3B), but Egger's test was not significant (*P* > 0.1). The incidence of LOS showed both funnel plot asymmetry and a significant Egger's test (*P* < 0.1), indicating possible publication bias (Figure 3C).

### 3.5 Subgroup analysis

A subgroup analysis was conducted to investigate the sources of heterogeneity. Table 2 provides a summary of the subgroup prevalence of sepsis among very preterm infants in China. Sepsis was categorized based on region, sample size, sample source, and definition. The results of the heterogeneity tests were statistically significant (*P* < 0.001) across all subgroups. At the regional level, the prevalence of unclassified sepsis was 27.15% (95% CI: 17.59%‒37.91%), while the prevalence of EOS in the mixed region was 1.41% (95% CI: 1.27%‒1.55%). Regarding sample size, participants were divided into subgroups of fewer than 1,000 and more than 1,000, following a common threshold from previous studies to distinguish between smaller and larger sample sizes. The incidence of unclassified sepsis in studies with ≤1,000 participants was 21.68% (95% CI: 11.13%‒34.50%), compared to 10.86% (95% CI: 3.14%‒22.44%) for EOS in studies with more than 1,000 participants. With respect to sample sources, single-center studies reported an incidence of unclassified sepsis at 21.86% (95% CI: 14.36%‒30.42%), whereas multi-center studies reported an incidence of LOS at 11.27% (95% CI: 8.25%‒14.71%). Based on the definition of sepsis, 2.30% (95% CI: 1.13%‒3.80%) of EOS cases were culture-proven, whereas 23.34% (95% CI: 12.80%‒35.90%) were identified as either culture-proven or clinical EOS.

**Table 2:** Subgroup summary incidence of sepsis among very preterm infants in China.

Sepsis type	Subgroup	Included studies (*n*)	Rate (%) (CI: 95%)	*I*^2^, *P* value
By region				
Unclassified sepsis	North	7	16.07 (10.70, 22.27)	*I*^2^ 96.36, *P* < 0.001
	Sourth	4	27.15 (17.59, 37.91)	*I*^2^ 98.20, *P* < 0.001
	Mixed	7	11.58 (6.17, 18.39)	*I*^2^ 99.83, *P* < 0.001
EOS	North	8	24.26 (12.56, 38.33)	*I*^2^ 99.79, *P* < 0.001
	Sourth	8	16.71 (9.44, 25.49)	*I*^2^ 97.01, *P* < 0.001
	Mixed	3	1.41 (1.27, 1.55)	*I*^2^ 0.00, *P* = 0.74
LOS	North	5	22.36 (16.95, 28.28)	*I*^2^ 96.62, *P* < 0.001
	South	10	14.54 (10.17, 19.51)	*I*^2^ 96.21, *P* < 0.001
	Mixed	2	4.87 (0.69, 12.47)	*I*^2^ 98.30, *P* < 0.001
By sample size				
Unclassified sepsis	>1,000	12	14.28 (9.19, 20.27)	*I*^2^ 99.80, *P* < 0.001
	≤1,000	6	21.68 (11.13, 34.50)	*I*^2^ 99.80, *P* < 0.001
EOS	>1,000	7	10.86 (3.14, 22.44)	*I*^2^ 99.92, *P* < 0.001
	≤1,000	12	19.79 (10.23, 31.50)	*I*^2^ 98.51, *P* < 0.001
LOS	<1,000	6	11.60 (9.27, 14.15)	*I*^2^ 96.90, *P* < 0.001
	≤1,000	11	18.87 (10.15, 29.43)	*I*^2^ 98.02, *P* < 0.001
By sample sources				
Unclassified sepsis	Single-center	7	21.86 (14.36, 30.42)	*I*^2^ 93.11, *P* < 0.001
	Multicenter	11	13.56 (8.39, 19.73)	*I*^2^ 99.81, *P* < 0.001
EOS	Single-center	9	21.83 (12.19, 33.29)	*I*^2^ 96.80, *P* < 0.001
	Multicenter	10	11.82 (4.71, 21.58)	*I*^2^ 99.89, *P* < 0.001
LOS	Single-center	9	20.99 (12.28, 31.24)	*I*^2^ 96.88, *P* < 0.001
	Multicenter	8	11.27 (8.25, 14.71)	*I*^2^ 98.26, *P* < 0.001
By sepsis definition				
Unclassified sepsis	Combined sepsis^a^	10	17.68 (10.06, 26.89)	*I*^2^ 99.72, *P* < 0.001
	Culture-proven sepsis	5	9.85 (8.39, 11.40)	*I*^2^ 95.45, *P* < 0.001
EOS	combined sepsis^a^	12	23.34 (12.80, 35.90)	*I*^2^ 99.84, *P* < 0.001
	Culture-proven sepsis	5	2.30 (1.13, 3.80)	*I*^2^ 93.24, *P* < 0.001
LOS	combined sepsis^a^	9	15.19 (11.60, 19.17)	*I*^2^ 96.63, *P* < 0.001
	Culture-proven sepsis	5	7.29 (9.61, 15.35)	*I*^2^ 96.46, *P* < 0.001

EOS, eary-onset sepsis; LOS, let-onset sepsis; M, mixed region of China; N, north of China; NR, not reported; S, south of China.

^a^ The combined data of culture-proven sepsis and clinical sepsis.

### 3.6 Sensitivity analysis

**Figure 4:**
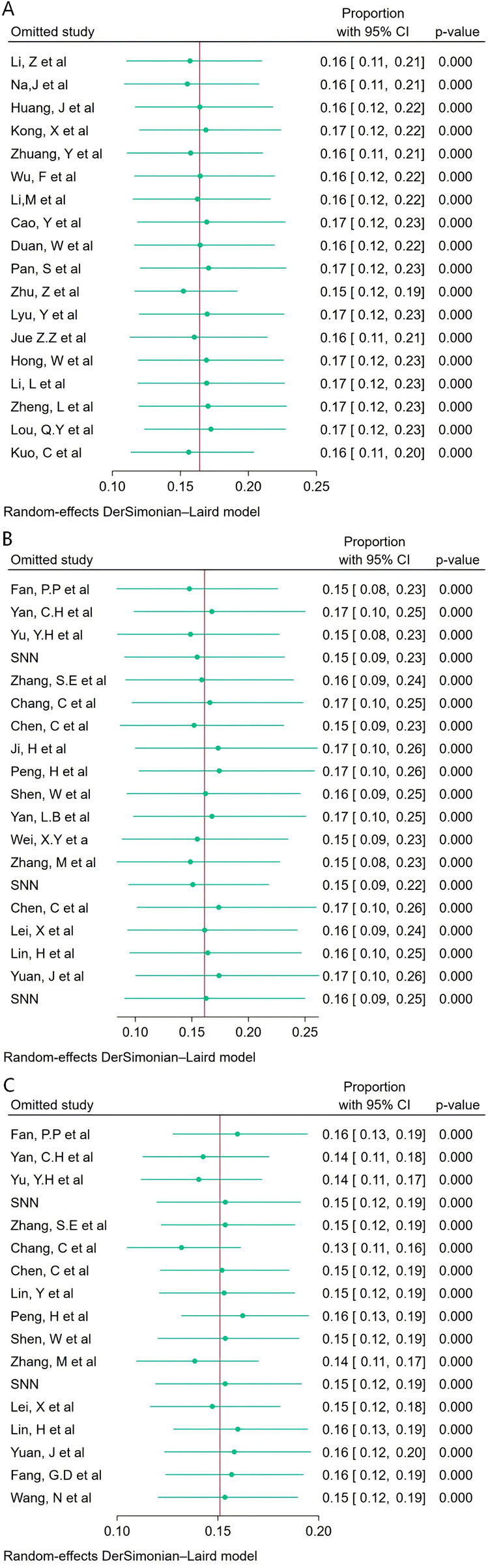
Sensitivity analysis of unclassified sepsis **(A)**, EOS **(B)** and LOS **(C)**.

A leave-one-out sensitivity analysis using the DerSimonian–Laird random-effects model was performed. After sequential omission of individual studies, the pooled incidence of unclassified sepsis (Figure 4A), EOS (Figure 4B), and LOS (Figure 4C) remained highly consistent with their respective overall effect estimates, with all *P* values <0.001, indicating stable and reliable results not overly influenced by any single study.

### 3.7 Factors associated with EOS among VPIs in China

**Figure 5:**
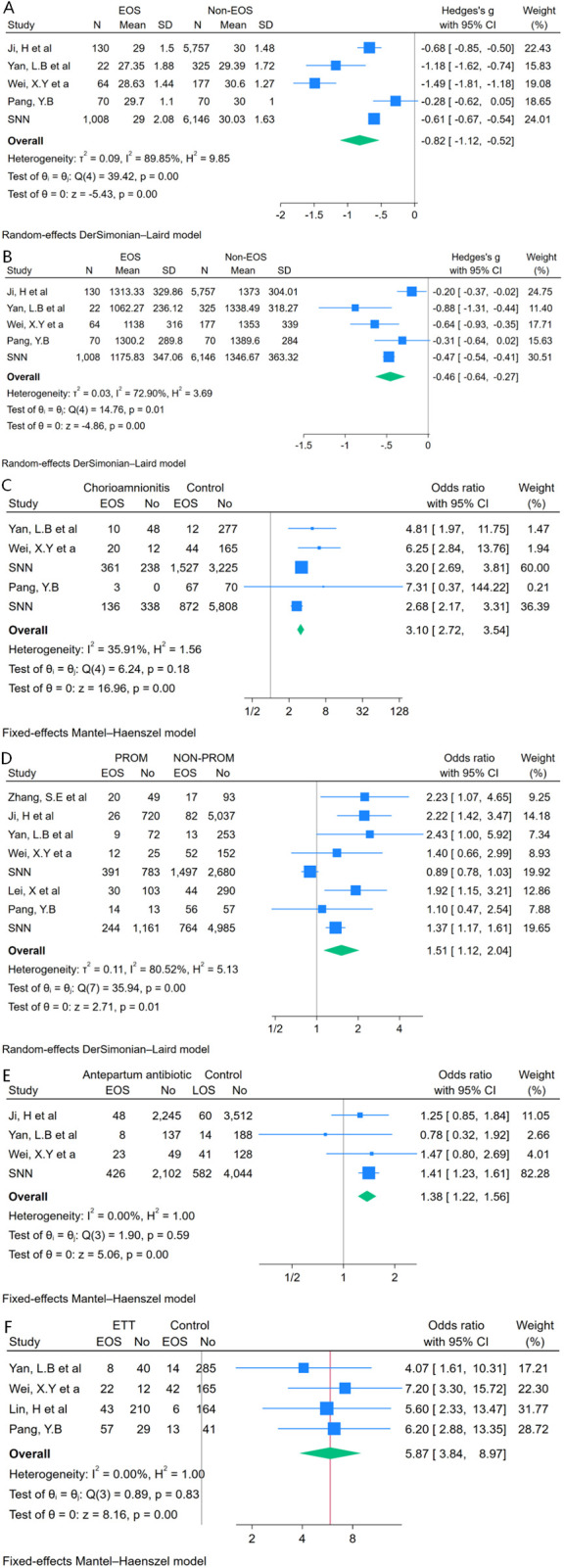
Factors associated with EOS among VPIs in China: lower gestational age **(A)**, lower birth weight **(B)**, chorioamnionitis **(C)**, premature rupture of membranes (PROM) **(D)**, antenatal antibiotics **(E)**, and endotracheal intubation **(F)**.

Due to the distinct influencing factors associated with EOS and LOS, a meta-analysis of risk factors for unclassified sepsis was not feasible. Furthermore, the insufficient number of studies examining LOS risk factors precluded the possibility of conducting a meta-analysis for this category. Consequently, this study exclusively conducted a meta-analysis on the risk factors for EOS. The analysis identified several significant risk factors, including lower gestational age [Hedges’ *g* = −0.82, 95% CI:(−1.12, −0.52)] (Figure 5A), lower birth weight [Hedges’ *g* = −0.46, 95% CI:(−0.64, −0.27)] (Figure 5B), chorioamnionitis (OR 3.10, 95% CI: 2.72–3.54) (Figure 5C), premature rupture of membranes (PROM) (OR 1.51, 95% CI: 1.12–2.04) (Figure 5D), antenatal antibiotic administration (OR 1.38, 95% CI: 1.22–1.56) (Figure 5E), and endotracheal intubation (OR 5.87, 95% CI: 3.84–8.97) (Figure 5F).

## 4 Discussion

This review and meta-analysis assessed the incidence of sepsis and its risk factors in VPIs in China by analyzing 43 studies. It identified six key risk factors for early-onset sepsis: lower gestational age, lower birth weight, chorioamnionitis, PROM, antenatal antibiotics, and endotracheal intubation.

### 4.1 Magnitude of neonatal sepsis

This systematic review represents the first comprehensive assessment of the overall burden of neonatal sepsis among VPIs in China. The incidence rates identified were 16.41% (95% CI: 11.80%‒21.62%) for unclassified sepsis, 16.11% (95% CI: 9.52%‒24.01%) for EOS, and 15.10% (95% CI: 12.07%‒18.40%) for LOS. The blood culture positivity rate for neonatal sepsis remains generally low, a phenomenon attributed to factors such as maternal and fetal antibiotic exposure, low-colony-count neonatal bacteremia, insufficient blood volume inoculation from neonates, and relatively slow turnaround times (56). Current evidence from systematic reviews indicates that blood cultures have a sensitivity of only 20.4% for detecting EOS (57). Our systematic review identified a culture-confirmed EOS incidence of 2.3% (95% CI: 1.13%‒3.80%) among VPIs. This finding is essentially consistent with reported rates of 3.6% in South Korea (58) and 1.35% in the United States (59). However, the overall incidence of culture-confirmed/clinical EOS in China's VPIs has reached 23.34% (95% CI 12.80%‒35.90%), highlighting diagnostic challenges. The clinical identification of neonatal sepsis is very challenging because of vague and nonspecific clinical signs (e.g., apnea, tachypnea, feeding intolerance and lethargy) (60). This diagnostic dilemma directly impacts therapeutic decision-making—clinicians frequently adopt a “treat upon suspicion” approach to avoid missed diagnoses (61), while the lack of reliable etiological evidence simultaneously leads to over-treatment (62). These findings underscore the need for continuous optimization of early diagnostic methods and improved risk-stratified management protocols. In recent years, multiple strategies have been proposed to address these challenges. Implementing routine antimicrobial stewardship programs in neonatal units can help optimize antibiotic use and reduce the risk of antimicrobial resistance (63). Recent studies indicate that umbilical cord blood cultures may improve pathogen detection rates (64), and efforts have been made to reduce the use of antibiotics for preterm babies at low risk of EOS (65–67). Furthermore, proteomics and metabolomics may contribute to precision diagnosis and personalized therapeutic strategies for neonatal sepsis (68, 69).

LOS occurs in 15.10% (95% CI 12.07%‒18.40%) of VPIs in China, exceeding the 8.9% rate reported in the VON database (70) but aligning with international data (10.8%–17.4%) (71–73). LOS carries significant mortality risks, with survivors demonstrating increased susceptibility to severe sequelae, including bronchopulmonary dysplasia, neurodevelopmental impairments (74, 75), and technology-dependent chronic conditions (e.g., home oxygen therapy, tracheostomy, and gastrostomy) (70). The dual challenges of improving survival rates among extremely preterm infants and the increasing prevalence of drug-resistant strains have intensified the clinical complexities of LOS management. While early identification of suspected cases remains critical for prognosis improvement, current practices lack validated LOS risk stratification tools. Recent studies demonstrate that monocyte distribution width (MDW), especially when combined with PCT, improves diagnostic accuracy for neonatal sepsis management (76). Concurrently, machine learning-based technologies provide objective, real-time decision support by analyzing routine NICU monitoring data, including vital signs such as heart rate, respiratory rate, and oxygen saturation. These innovative diagnostic approaches are advancing neonatal sepsis management toward greater precision and efficiency (77–79).

This systematic review revealed that neonatal sepsis remains a significant health burden among Chinese VPIs, with an incidence of 16.41%. Subgroup analyses revealed substantial heterogeneity in sepsis incidence, with higher rates in northern regions, single-center studies, and smaller cohorts. The wide 95% confidence intervals indicate significant variability across studies, likely reflecting regional disparities in neonatal healthcare resources. Furthermore, methodological variations—including differences in population selection, diagnostic criteria, and data collection—may influence reported incidence rates. Inconsistencies in sepsis definitions and diagnostic approaches across studies may also affect comparability. These results highlight the necessity for future large-scale, multicenter, high-quality studies to assess the burden of neonatal sepsis in Chinese VPIs more accurately and provide robust evidence for targeted prevention and control strategies.

### 4.2 Factors associated with EOS

The risk of EOS increases with decreasing gestational age (GA) or birth weight (BW), with GA being the strongest predictive indicator for EOS (36, 80). This meta-analysis confirmed that both GA [Hedges’ *g* = −0.82, 95% CI (−1.12, −0.52)] and BW [Hedges’ *g* = −0.46, 95% CI (−0.64, −0.27)] are perinatal risk factors for EOS. A recent clinical study further confirmed that gestational age and birth weight were inversely associated with susceptibility to neonatal sepsis, which is consistent with the findings of the present study (81). Currently, the standard clinical practice for preterm infants of lower gestational age (typically VPIs) involves immediate postnatal evaluation and low-threshold antibiotic treatment (82). While this strategy has effectively reduced the incidence of EOS, it has also led to increased unnecessary antibiotic exposure. In view of this situation, implementing close monitoring with routine intensive care surveillance rather than empirical antibiotic therapy may represent a feasible solution to address this clinical dilemma.

Numerous studies have confirmed that chorioamnionitis (CA) is a key risk factor for EOS in preterm infants (83, 84), potentially mediated by the immunomodulatory role of prenatal infection/inflammation (84). This systematic review confirms that CA is a significant risk factor for EOS in VPIs (OR 3.10, 95% CI 2.72‒3.54). For VPIs born to mothers with CA, our research supports the recommendation of performing infection assessments and initiating empirical antibiotic therapy until culture results are available. In contrast, some studies have shown that the absence of CA can be used as a factor to identify preterm infants with a lower risk of EOS, thus preventing unnecessary antibiotic exposure (65). This risk stratification strategy is helpful for optimizing early antibiotic decision-making for VPIs.

PROM serves both as a potential marker of intrauterine infection and a mechanical compromise of the fetal membrane barrier, enabling pathogenic invasion (85). These dual pathological mechanisms establish PROM as a major risk factor for EOS (86). This systematic review confirmed that PROM increases the risk of EOS in VPIs (OR 1.51, 95% CI: 1.12–2.04). While term infants demonstrate a linear correlation between the duration of membrane rupture and EOS risk (87), the link between PROM and EOS risk in preterm infants is more complex and is influenced not only by its occurrence or duration but also by gestational age, clinical chorioamnionitis, and intrapartum antibiotic administration (80). Future research should develop a multiparameter risk model incorporating gestational age, inflammatory markers, antibiotic use, and microbiological data to predict PROM-associated EOS in VPIs, optimizing clinical management.

Antenatal antibiotic use is primarily indicated for treating maternal chorioamnionitis or as prophylaxis on the basis of positive GBS screening. While antenatal antibiotic prophylaxis is considered the standard for reducing EOS risk in term infants (88), its potential adverse effects—including antimicrobial resistance and alterations in microbial profiles—are increasingly recognized (89). Antenatal antibiotic exposure can lead to dysbiosis of the maternal vaginal and neonatal intestinal microbiota, and this microbial imbalance may further increase the risk of EOS (90). Our study revealed a modest association between antenatal antibiotic exposure and EOS risk in Chinese VPIs (OR 1.38, 95% CI: 1.22‒1.56), which may be influenced by population-specific characteristics, GBS screening coverage, and antibiotic stewardship protocols. Future efforts should optimize protocols by incorporating local pathogen distributions and resistance surveillance data while enforcing strict indications for antenatal antibiotics and establishing institutional EOS pathogen monitoring systems to guide clinical practice.

Owing to immature lung development, VPIs often require respiratory support after birth. Endotracheal intubation plays a critical role in delivery room resuscitation for these infants, with a reported intubation rate of 26.3%–37% in China (20, 91). However, as an invasive procedure, intubation may damage mucosal barriers and significantly increase the risk of EOS (OR 5.87, 95% CI: 3.84–8.97). Current evidence increasingly supports prioritizing noninvasive ventilation as the initial respiratory support strategy (92–94). With increasing understanding of normal oxygen saturation levels in the first minutes after birth (95, 96), avoiding excessive endotracheal intubation and mechanical ventilation for VPIs at birth may become possible.

In the clinical management of sepsis in VPIs, we believe that implementing individualized risk assessment and evidence-based management is crucial. Currently, research on predictive models specifically for VPIs is insufficient, particularly in low- and middle-income countries (97). Future studies could integrate risk factors and region-specific pathogen profiles, among other key variables, to develop more targeted predictive models. This would provide more reliable clinical decision-making support for the precise prevention and management of sepsis in VPIs.

## 5 Strengths and limitations of the study

This study represents the first systematic review and meta-analysis on the incidence and risk factors for sepsis in Chinese VPIs, addressing a critical gap in regional evidence. By synthesizing the available evidence from both Chinese- and English-language literature, this study provides China-specific epidemiological evidence on neonatal sepsis in VPIs for an international audience. With a large sample size (>138,613 cases) and subgroup analyses, the findings enhance statistical power and clinical applicability.

This study is subject to several limitations. First, to comprehensively assess the disease burden of sepsis in very preterm infants in China, we included all eligible studies published in both Chinese and English. However, the diagnostic criteria for sepsis were not fully consistent across studies, resulting in a certain degree of heterogeneity. Although the latest clinical practice guideline was released in China in 2024, it was issued relatively late, and no published studies using this updated guideline as the diagnostic basis were identified by the literature search cutoff date of this study. Therefore, the diagnostic criteria based on this guideline could not be reflected in the present analysis. Second, there was significant statistical heterogeneity among the included studies. Despite conducting extensive subgroup analyses, the high degree of heterogeneity suggests the presence of unmeasured confounding factors, such as variations in resource allocation and clinical care standards across study centers. The inability to quantify the impact of these factors may limit the generalizability of the pooled estimates. Third, the presence of funnel plot asymmetry and the results of Egger's test (*P* < 0.1) indicate substantial publication bias, which could have led to an overestimation of effect sizes. Fourth, the majority of the primary studies included in the analysis reported unadjusted ORs without accounting for potential confounding factors. This reliance on unadjusted effect estimates may have introduced residual confounding bias into our risk factor analysis, thereby diminishing the reliability of the findings. Furthermore, the limited number of eligible studies precluded the possibility of conducting a meta-analysis for LOS and certain risk factors for EOS, which, to some extent, constrained the comprehensiveness of the conclusions.

## 6 Conclusion

This study revealed that the incidence of sepsis in Chinese VPIs was 16.41% (95% CI: 11.80%‒21.62%) for unclassified sepsis, 16.11% (95% CI: 9.52%‒24.01%) for EOS, and 15.10% (95% CI: 12.07%‒18.40%) for LOS. Notably, the overall clinical sepsis rate (based on clinical diagnosis ± culture) significantly exceeds the culture-confirmed EOS rate, highlighting diagnostic challenges and therapeutic dilemmas in this vulnerable population. The key risk factors for EOS include lower gestational age, lower birth weight, chorioamnionitis, PROM, antenatal antibiotic exposure, and ETT. These findings provide critical evidence for developing risk prediction models and stratified management strategies.
