# Supplementary material for: Prevalence and risk factors for neonatal sepsis among very preterm infants in China: a systematic review and meta-analysis
Source: Front Pediatr. 2026 Apr 1;14:1815128. doi: 10.3389/fped.2026.1815128 (PMC13079635; doi:10.3389/fped.2026.1815128)
Supplement: Supplementary file 3 [file supplementaryfile3.docx]

2025-3-31

**Pubmed**

| # | searches | resultes |
| --- | --- | --- |
| 1 | ("Infant, Premature"[Mesh] OR "preterm infant*"[Title/Abstract] OR "premature infant*"[Title/Abstract] OR "preterm neonat*"[Title/Abstract] OR "premature neonat*"[Title/Abstract] OR "Infant, Extremely Premature"[Mesh] OR "extremely preterm infant*"[Title/Abstract] OR "Infant, Premature, Diseases"[Mesh] OR "preterm birth*"[Title/Abstract]) OR "Infant, extremely low birth weight"[MeSH] OR "very preterm infants"[Title/Abstract] | 142,039 |
| 2 | "Sepsis"[MeSH Terms] OR "Sepsis"[Title/Abstract] OR "septic syndrome"[Title/Abstract] OR "Systemic Inflammatory Response Syndrome"[MeSH Terms] OR "SIRS"[Title/Abstract] OR "septic shock"[Title/Abstract] OR "severe sepsis"[Title/Abstract] | 248,735 |
| 3 | "bloodstream infection*"[Title/Abstract] OR "BSI"[Title/Abstract] OR "Bacteremia"[MeSH Terms] OR "Bacteremia"[Title/Abstract] OR "bacteraemia"[Title/Abstract] OR "septicemia"[Title/Abstract] OR "septicaemia"[Title/Abstract] OR "blood infection*"[Title/Abstract] OR "blood borne infection*"[Title/Abstract] | 89,705 |
| 4 | China[Title/Abstract] OR Chinese[Title/Abstract] OR Guangxi[Title/Abstract] OR "inner Mongolia"[Title/Abstract] OR Ningxia[Title/Abstract] OR Tibet[Title/Abstract] OR Xizang[Title/Abstract] OR Xinjiang[Title/Abstract] OR Heilongjiang[Title/Abstract] OR Jilin[Title/Abstract] OR Liaoning[Title/Abstract] OR Hebei[Title/Abstract] OR Shanxi[Title/Abstract] OR Shandong[Title/Abstract] OR Shaanxi[Title/Abstract] OR Gansu[Title/Abstract] OR Qinghai[Title/Abstract] OR Sichuan[Title/Abstract] OR Hubei[Title/Abstract] OR Hunan[Title/Abstract] OR Henan[Title/Abstract] OR Anhui[Title/Abstract] OR Zhejiang[Title/Abstract] OR Jiangsu[Title/Abstract] OR Guangdong[Title/Abstract] OR Jiangxi[Title/Abstract] OR Fujian[Title/Abstract] OR Guizhou[Title/Abstract] OR Yunnan[Title/Abstract] OR Hainan[Title/Abstract] OR Han[Title/Abstract] OR Hui[Title/Abstract] OR Mongolia[Title/Abstract] OR Zhuang[Title/Abstract] OR Taiwan[Title/Abstract] OR "Hong Kong"[Title/Abstract] OR Macau[Title/Abstract] | 712,304 |
| 5 | #1 AND (#2 OR #3) AND #4 | [1](https://pubmed.ncbi.nlm.nih.gov/?term=(("Incidence"[Mesh])+OR+("Epidemiology"[Mesh]+AND+"incidence"[Title/Abstract])+OR+("Disease+Outbreaks"[Mesh]+AND+"incidence"[Title/Abstract])+OR+incidence[Title/Abstract]+OR+"occurrence+rate"[Title/Abstract]+OR+"event+rate"[Title/Abstract]+OR+"cumulative+incidence"[Title/Abstract]+OR+"incidence+rate"[Title/Abstract]+OR+"incidence+density"[Title/Abstract])%0D%0A&sort=)93 |

**Embase**

| # | searches | resultes |
| --- | --- | --- |
| 1 | 'prematurity'/exp OR 'prematurity' OR 'preterm infant*':ti,ab OR 'premature infant*':ti,ab OR 'preterm neonat*':ti,ab OR 'premature neonat*':ti,ab OR 'extremely preterm infant'/exp OR 'extremely preterm infant' OR 'extremely preterm infant*':ti,ab OR 'prematurity disease'/exp OR 'prematurity disease' OR 'preterm birth*':ti,ab OR 'extremely low birth weight infant'/exp OR 'extremely low birth weight infant' OR 'very preterm infant*':ti,ab | 204,725 |
| 2 | 'sepsis'/exp OR 'sepsis' OR 'sepsis':ti,ab OR 'septic syndrome':ti,ab OR 'systemic inflammatory response syndrome'/exp OR 'systemic inflammatory response syndrome' OR 'sirs':ti,ab OR 'septic shock':ti,ab OR 'severe sepsis':ti,ab | 455,445 |
| 3 | 'bloodstream infection*':ti,ab OR 'bsi':ti,ab OR 'bacteremia'/exp OR 'bacteremia' OR 'bacteremia':ti,ab OR 'bacteraemia':ti,ab OR 'septicemia':ti,ab OR 'septicaemia':ti,ab OR 'blood infection*':ti,ab OR 'blood borne infection*':ti,ab | [130,23](https://www-embase-com-443.webvpn.cams.tsgvip.top/)3 |
| 4 | 'china':ti,ab,kw OR 'chinese':ti,ab,kw OR 'guangxi':ti,ab,kw OR 'inner mongolia':ti,ab,kw OR 'ningxia':ti,ab,kw OR 'tibet':ti,ab,kw OR 'xizang':ti,ab,kw OR 'xinjiang':ti,ab,kw OR 'heilongjiang':ti,ab,kw OR 'jilin':ti,ab,kw OR 'liaoning':ti,ab,kw OR 'hebei':ti,ab,kw OR 'shanxi':ti,ab,kw OR 'shandong':ti,ab,kw OR 'shaanxi':ti,ab,kw OR 'gansu':ti,ab,kw OR 'qinghai':ti,ab,kw OR 'sichuan':ti,ab,kw OR 'hubei':ti,ab,kw OR 'hunan':ti,ab,kw OR 'henan':ti,ab,kw OR 'anhui':ti,ab,kw OR 'zhejiang':ti,ab,kw OR 'jiangsu':ti,ab,kw OR 'guangdong':ti,ab,kw OR 'jiangxi':ti,ab,kw OR 'fujian':ti,ab,kw OR 'guizhou':ti,ab,kw OR 'yunnan':ti,ab,kw OR 'hainan':ti,ab,kw OR 'han':ti,ab,kw OR 'hui':ti,ab,kw OR 'mongolia':ti,ab,kw OR 'zhuang':ti,ab,kw OR 'taiwan':ti,ab,kw OR 'hong kong':ti,ab,kw OR 'macau':ti,ab,kw | 861,495 |
| 7 | #1 AND (#2 OR #3) AND #4 | 355 |

**Scopus**

| # | searches | resultes |
| --- | --- | --- |
| 1 | TITLE-ABS-KEY ( "preterm infant*" OR "premature infant*" OR "preterm neonat*" OR "premature neonat*" OR "extremely preterm infant*" OR "preterm birth*" OR "very preterm infant*" OR "extremely low birth weight" ) | 108,087 |
| 2 | TITLE-ABS-KEY ( "neonatal sepsis" OR "sepsis" OR "septic syndrome" OR "systemic inflammatory response syndrome" OR "SIRS" OR "septic shock" OR "severe sepsis" OR "bloodstream infection*" OR "BSI" OR "bacteremia" OR "bacteraemia" OR "septicemia" OR "septicaemia" OR "blood infection*" OR "blood borne infection*" ) | 464,278 |
| 3 | China OR Chinese OR Guangxi OR "inner Mongolia" OR Ningxia OR Tibet OR Xizang OR Xinjiang OR Heilongjiang OR Jilin OR Liaoning OR Hebei OR Shanxi OR Shandong OR Shaanxi OR Gansu OR Qinghai OR Sichuan OR Hubei OR Hunan OR Henan OR Anhui OR Zhejiang OR Jiangsu OR Guangdong OR Jiangxi OR Fujian OR Guizhou OR Yunnan OR Hainan OR Han OR Hui OR Mongolia OR Zhuang OR Taiwan OR Hong Kong OR Macau | 3,466,929 |
| 4 | #1 AND #2 AND #3 | 313 |

**Web of science**

| # | searches | resultes |
| --- | --- | --- |
| 1 | TS=("preterm infant*" OR "premature infant*" OR "preterm neonat*" OR "premature neonat*" OR "extremely preterm" OR "preterm birth*" OR "very preterm" OR "extremely low birth weight") | 138,803 |
| 2 | TS=("neonatal sepsis" OR sepsis OR "septic syndrome" OR "systemic inflammatory response" OR SIRS OR "septic shock" OR "severe sepsis" OR "bloodstream infection*" OR BSI OR bacteremia OR bacteraemia OR septicemia OR septicaemia OR "blood infection*" OR "blood borne infection*") | 420,272 |
| 3 | TS=(China OR Chinese OR Guangxi OR "inner Mongolia" OR Ningxia OR Tibet OR Xizang OR Xinjiang OR Heilongjiang OR Jilin OR Liaoning OR Hebei OR Shanxi OR Shandong OR Shaanxi OR Gansu OR Qinghai OR Sichuan OR Hubei OR Hunan OR Henan OR Anhui OR Zhejiang OR Jiangsu OR Guangdong OR Jiangxi OR Fujian OR Guizhou OR Yunnan OR Hainan OR Han OR Hui OR Mongolia OR Zhuang OR Taiwan OR Hong Kong OR Macau) | 2,854,019 |
| 4 | #1 AND #2 AND #3 | 463 |

**CNKI**

| # | searches | resultes |
| --- | --- | --- |
| 1 | SU=(极早产儿+超早产儿+超低出生体重儿+32周以下) and SU=(败血症+脓毒) and SU=(发生率+发生+流行病学+患病率+影响因素+危险因素) | 119 |

**Wangfang**

| # | searches | resultes |
| --- | --- | --- |
| 1 | 主题:("极早产儿" or "超早产儿" or "超低出生体重儿" or "32周以下"or "<32周") and 主题:( "败血症" or "脓毒") and 主题:("发生率" or "发生" or "流行病学" or "患病率") and 主题:("影响因素" or "危险因素") | 166 |

**CSTJ**

| # | searches | resultes |
| --- | --- | --- |
| 1 | M=(极早产儿 or 超早产儿 or 超低出生体重儿 or 32周以下 or <32周) | 1861 |
| 2 | M=(败血症 or 脓毒 or 血流感染) | 36726 |
| 3 | M=(发生率 or 发生 or 流行病学 or 患病率 or 影响因素 or 危险因素) | 1,048,041 |
| 4 | #1 AND #2 AND #3 | 16 |

**CBM**

| # | searches | resultes |
| --- | --- | --- |
| 1 | \| "极早产儿"[常用字段:智能] OR "超早产儿"[常用字段:智能] OR "超低出生体重儿"[常用字段:智能] OR "＜32周"[常用字段:智能] OR "32周以下"[常用字段:智能] \| \| --- \| | [5872](javascript:historyLink('"%E6%9E%81%E6%97%A9%E4%BA%A7%E5%84%BF"[%E5%B8%B8%E7%94%A8%E5%AD%97%E6%AE%B5:%E6%99%BA%E8%83%BD] OR "%E8%B6%85%E6%97%A9%E4%BA%A7%E5%84%BF"[%E5%B8%B8%E7%94%A8%E5%AD%97%E6%AE%B5:%E6%99%BA%E8%83%BD] OR "%E8%B6%85%E4%BD%8E%E5%87%BA%E7%94%9F%E4%BD%93%E9%87%8D%E5%84%BF"[%E5%B8%B8%E7%94%A8%E5%AD%97%E6%AE%B5:%E6%99%BA%E8%83%BD] OR "%EF%BC%9C32%E5%91%A8"[%E5%B8%B8%E7%94%A8%E5%AD%97%E6%AE%B5:%E6%99%BA%E8%83%BD] OR "32%E5%91%A8%E4%BB%A5%E4%B8%8B"[%E5%B8%B8%E7%94%A8%E5%AD%97%E6%AE%B5:%E6%99%BA%E8%83%BD]')) |
| 2 | "败血症"[常用字段:智能] OR "脓毒"[常用字段:智能] OR "血流感染"[常用字段:智能] | [59072](javascript:historyLink('"%E8%B4%A5%E8%A1%80%E7%97%87"[%E5%B8%B8%E7%94%A8%E5%AD%97%E6%AE%B5:%E6%99%BA%E8%83%BD] OR "%E8%84%93%E6%AF%92"[%E5%B8%B8%E7%94%A8%E5%AD%97%E6%AE%B5:%E6%99%BA%E8%83%BD] OR "%E8%A1%80%E6%B5%81%E6%84%9F%E6%9F%93"[%E5%B8%B8%E7%94%A8%E5%AD%97%E6%AE%B5:%E6%99%BA%E8%83%BD]')) |
| 3 | "发生率"[常用字段:智能] OR "发生"[常用字段:智能] OR "流行病学"[常用字段:智能] OR "患病率"[常用字段:智能] | [2658084](javascript:historyLink('"%E5%8F%91%E7%94%9F%E7%8E%87"[%E5%B8%B8%E7%94%A8%E5%AD%97%E6%AE%B5:%E6%99%BA%E8%83%BD] OR "%E5%8F%91%E7%94%9F"[%E5%B8%B8%E7%94%A8%E5%AD%97%E6%AE%B5:%E6%99%BA%E8%83%BD] OR "%E6%B5%81%E8%A1%8C%E7%97%85%E5%AD%A6"[%E5%B8%B8%E7%94%A8%E5%AD%97%E6%AE%B5:%E6%99%BA%E8%83%BD] OR "%E6%82%A3%E7%97%85%E7%8E%87"[%E5%B8%B8%E7%94%A8%E5%AD%97%E6%AE%B5:%E6%99%BA%E8%83%BD]')) |
| 4 | "影响因素"[常用字段:智能] OR "危险因素"[常用字段:智能] | [526539](javascript:historyLink('"%E5%BD%B1%E5%93%8D%E5%9B%A0%E7%B4%A0"[%E5%B8%B8%E7%94%A8%E5%AD%97%E6%AE%B5:%E6%99%BA%E8%83%BD] OR "%E5%8D%B1%E9%99%A9%E5%9B%A0%E7%B4%A0"[%E5%B8%B8%E7%94%A8%E5%AD%97%E6%AE%B5:%E6%99%BA%E8%83%BD]')) |
| 5 | #1 AND #2 AND (#3 OR #4) | 318 |

In total=1943

Find duplication=530
