# Supplementary material for: Prevalence and risk factors for neonatal sepsis among very preterm infants in China: a systematic review and meta-analysis
Source: Front Pediatr. 2026 Apr 1;14:1815128. doi: 10.3389/fped.2026.1815128 (PMC13079635; doi:10.3389/fped.2026.1815128)
Supplement: Supplementary file 5 [file supplementaryfile5.docx]

**Supplementary Material 5 EOS-related risk factors with nonsignificant pooled effect sizes**

| **Risk factor** | **OR (95%CI)** | ***I^2^*,*P*Value** |
| --- | --- | --- |
| Antenatal steroid | 1.38 (0.88, 2.18) | *I^2^* 68.69, *P* =0.16 |
| GDM | 1.05 (0.9, 1.22) | *I^2^* 0.00, *P* =0.56 |
| HDCP | 0.93(0.67, 1.29) | *I^2^* 90.38, *P* =0.65 |
| IVF-ET | 1.26 (0.69, 2.31) | *I^2^*66.79, *P* =0.45 |
| Male | 1.03 (0.91, 1.17) | *I^2^*36.54, *P* =0.61 |
| SGA | 1.06 (0.61, 1.87) | *I^2^* 65.02, *P* =0.83 |
| Multiple birth | 0.90 (0.78, 1.05) | *I^2^* 0.00, *P* =0.17 |
| Mother’s age | 0.05（-0.03，0.13） | *I^2^*12.23, *P* =0.23 |

Note: GDM: Gestational Diabetes Mellitus; HDCP: Hypertensive Disorder Complicating Pregnancy; IVF-ET: In Vitro Fertilization and Embryo Transfer; SGA: Small for Gestational Age
